# Methylation of the leukocyte glucocorticoid receptor gene promoter in adults: associations with early adversity and depressive, anxiety and substance-use disorders

**DOI:** 10.1038/tp.2016.112

**Published:** 2016-07-05

**Authors:** A R Tyrka, S H Parade, E S Welch, K K Ridout, L H Price, C Marsit, N S Philip, L L Carpenter

**Affiliations:** 1 Mood Disorders Research Program and Laboratory for Clinical and Translational Neuroscience, Butler Hospital, Providence, RI, USA; 2 Department of Psychiatry and Human Behavior, Alpert Medical School of Brown University, Providence, RI, USA; 3 Bradley/Hasbro Children's Research Center, E. P. Bradley Hospital, East Providence, RI, USA; 4 Department of Pharmacology and Toxicology, Geisel School of Medicine at Dartmouth, Hanover, NH, USA; 5 Department of Epidemiology, Geisel School of Medicine at Dartmouth, Hanover, NH, USA; 6 Center for Neurorestoration and Neurotechnology, Providence VA Medical Center, Providence, RI, USA

## Abstract

Early adversity increases risk for developing psychopathology. Epigenetic modification of stress reactivity genes is a likely mechanism contributing to this risk. The glucocorticoid receptor (GR) gene is of particular interest because of the regulatory role of the GR in hypothalamic–pituitary–adrenal (HPA) axis function. Mounting evidence suggests that early adversity is associated with GR promoter methylation and gene expression. Few studies have examined links between GR promoter methylation and psychopathology, and findings to date have been mixed. Healthy adult participants (*N*=340) who were free of psychotropic medications reported on their childhood experiences of maltreatment and parental death and desertion. Lifetime depressive and anxiety disorders and past substance-use disorders were assessed using the Structured Clinical Interview for the Diagnostic and Statistical Manual of Mental Disorders, Fourth Edition. Methylation of exon 1_F_ of the GR gene (*NR3C1*) was examined in leukocyte DNA via pyrosequencing. On a separate day, a subset of the participants (*n*=231) completed the dexamethasone/corticotropin-releasing hormone (Dex/CRH) test. Childhood adversity and a history of past substance-use disorder and current or past depressive or anxiety disorders were associated with lower levels of *NR3C1* promoter methylation across the region as a whole and at individual CpG sites (*P*<0.05). The number of adversities was negatively associated with *NR3C1* methylation in participants with no lifetime disorder (*P*=0.018), but not in those with a lifetime disorder. GR promoter methylation was linked to altered cortisol responses to the Dex/CRH test (*P*<0.05). This study presents evidence of *reduced* methylation of *NR3C1* in association with childhood maltreatment and depressive, anxiety and substance-use disorders in adults. This finding stands in contrast to our prior work, but is consistent with emerging findings, suggesting complexity in the regulation of this gene.

## Introduction

Substantial evidence from animal models and human studies implicates impairments in glucocorticoid signaling in stress-related psychiatric disorders, such as major depressive disorder (MDD) and post-traumatic stress disorder (PTSD). Changes in glucocorticoid receptor (GR) number and function in the brain and in peripheral cells such as leukocytes have been shown with PTSD, MDD and early stress exposure,^1, 2, 3, 4, 5, 6, 7, 8^ and alterations of diurnal and reactive cortisol concentrations are seen in adults and children with these conditions.^9, 10, 11^ GR-mediated negative feedback has a critical role in dampening activity of the hypothalamic–pituitary–adrenal (HPA) axis so that changes in GR number or function can influence activity of this system and, consequently, the biological adaptation to stressful or traumatic experiences. Because childhood maltreatment and other adverse experiences are major risk factors for the development of psychopathology, including depressive, anxiety and substance-use disorders,^12, 13, 14, 15^ stress-induced alterations in glucocorticoid signaling may be a mechanism of these associations.

Expression of the human GR gene, *NR3C1*, is regulated by a variety of transcriptional and translational mechanisms.^16^ A growing area of research is aimed at determining whether early-life stress is associated with epigenetic changes to the promoter region of *NR3C1* that alter GR expression and potentially alter HPA-axis homeostasis and responses to stress. Epigenetic modifications to the genome allow for altered gene expression but do not change the DNA sequence and thus permit elaboration of the genome beyond what is determined by DNA base coding.^17, 18^ Methylation, involving the addition of a methyl group to DNA, is thought to be the most stable form of epigenetic alteration.^18, 19^ Typically, this occurs at sites where a cytosine nucleotide occurs next to a guanine nucleotide (CpG dinucleotides), although non-CpG methylation has been discovered in embryonic stem cells and in some adult brain, skeletal muscle, and hematopoietic cells.^20^ Methylation at promoter CpG sites can lead to alterations in chromatin architecture and inhibit transcription factor (TF) binding, often resulting in reduced gene expression. Consistent with this, genes such as *NR3C1* that are highly expressed typically have low levels of promoter methylation.^17, 21, 22^

Differential methylation has been shown to be associated with stress exposure early in life. Early studies in rodents with naturally occurring differences in maternal care showed that low levels of care were associated with greater methylation of the GR gene, specifically in the promoter region homologous to the human exon 1_F_.^23, 24^ The epigenetic effects of differential maternal care were associated with long-lasting effects on stress responses.^23, 24^ Subsequently, a number of studies in humans across the developmental spectrum have documented a relationship between early environmental exposures and increased methylation of the alternate first exons in the *NR3C1* promoter.^25, 26^

Beginning with prenatal exposure, several studies have demonstrated that exposure to maternal depression, anxiety or trauma during gestation is associated with greater levels of methylation of the 1_F_ region in DNA from placental tissue and umbilical cord blood.^27, 28, 29, 30, 31^ Childhood maltreatment was associated with increased *NR3C1* methylation in leukocyte DNA from children aged 11–14 years^32^ and in saliva DNA from preschool-aged children.^33^ Two studies of adolescents have also shown positive associations of methylation of the *NR3C1* promoter in peripheral blood DNA with prenatal exposure to intimate partner violence^34^ or traumatic experiences in childhood.^35^ The enduring effects of early stress are appreciated from evidence that alterations of *NR3C1* promoter methylation were seen in adults with a history of childhood adversity. In a preliminary study of healthy adults with no lifetime psychopathology, our group found positive associations of 1_F_ methylation with childhood parental loss and maltreatment that were not accounted for by recent perceived stress.^36^ Taken together, these findings suggest that altered *NR3C1* methylation is linked to early stress exposure, and thus might predispose to the development of psychopathology.

Early-life adversity is a known risk factor for psychopathology later in life,^12, 13, 14, 15^ and differential methylation of genes important to the regulation of stress, such as the GR, may be a mechanism of this effect. However, few studies have examined the relationship between *NR3C1* methylation, childhood adversity and psychopathology. We recently reported that methylation of saliva DNA *NR3C1* in association with maltreatment and other adversities in maltreated preschool-aged children mediated the link between early stress exposure and internalizing behaviors (that is, withdrawn, somatic, anxious and depressed).^37^ In a study of post-mortem brain from suicides, subjects with a history of childhood abuse had differential *NR3C1* promoter methylation compared with suicide completers without a history of childhood abuse and controls without a history of childhood abuse or suicide.^38^ Several investigations found altered methylation of sites in the *NR3C1* promoter region in those with psychopathology.^39, 40, 41, 42, 43, 44, 45, 46^ A few studies have examined effects of childhood adversity within the sample as a whole, with some finding no effect^39, 43, 44, 45^ and others finding an effect of early-life stress on increased *NR3C1* methylation.^41, 47, 48^ However, these have not specifically examined the differential contributions of early stress and psychopathology to *NR3C1* methylation. Moreover, most prior work in individuals with psychiatric disorders included participants on psychotropic medication, which can alter methylation levels.^48, 49, 50^

Another issue requiring further study is the direction of methylation effects in association with early stress and psychopathology. For early stress, most work has identified hypermethylation of *NR3C1* in the 1_F_ region or its rat homolog.^25^ However, a number of these studies reported lower levels of methylation in some of the 1_F_ CpG sites,^28, 32, 35, 38^ and examination of methylation at specific CpG sites may be important to understand the regulation of this gene.^51, 52^ For psychopathology, findings as to the direction of methylation effects have been mixed. Increased levels of *NR3C1* promoter methylation have been shown among children and adolescents with depression or internalizing problems;^46, 53^ however, a study of depressed outpatients documented decreased levels of methylation in comparison with controls.^42^ Increased methylation has been documented in borderline personality disorder^39, 41^ and in women with comorbid borderline personality disorder and bulimia nervosa.^43^ In contrast, several studies have demonstrated lower levels of *NR3C1* methylation in patients with PTSD as compared with healthy controls^45, 54^ and PTSD symptoms in men.^44^ Finally, recent research has shown that externalizing disorders and problems are also characterized by hypomethylation of *NR3C1.*^40, 55^

In order to examine these issues, the present study investigated methylation of the *NR3C1* 1_F_ promoter region in adults with and without a history of childhood adversity, and with and without a lifetime psychiatric diagnosis of depressive, anxiety or substance-use disorder.

## Materials and methods

### Participants

Three-hundred and forty adults, 213 women and 127 men, aged 18–65 (32.9±11.5) years, were recruited in a series of related studies using separate local, newspaper and internet advertisements directed toward healthy adults, adults with depression, individuals with a history of early parental loss and adults with a history of early-life stress. This sample did not include the participants from our prior study of childhood adversity and *NR3C1* methylation.^36^ The study was approved by the Butler Hospital Institutional Review Board, and, after complete description of the study to the participants, voluntary written informed consent was obtained.

Exclusions included prescription medication use other than oral contraceptives, reported acute or chronic medical illness, pregnancy, a history of brain injury or seizure disorder, night-shift work and current alcohol- or substance-use disorders because of possible effects on activity of the HPA axis. In addition, excluded were those with a lifetime history of bipolar disorder, psychotic disorder and obsessive–compulsive disorder. The subset of participants who completed the dexamethasone/corticotropin-releasing hormone (Dex/CRH) test (*n*=231) completed a physical examination, electrocardiogram and standard laboratory studies to further rule out acute or unstable medical illness. Oral contraceptives were allowed, with usage accounted for in analyses of cortisol concentrations.

### Assessment of diagnoses and symptoms

Axis I psychiatric diagnoses were assessed using the Structured Clinical Interview for the Diagnostic and Statistical Manual of Mental Disorders, Fourth Edition.^56^ The 30-item Inventory of Depressive Symptomatology, Self-Report version,^57^ the State-Trait Anxiety Inventory^58^ and the Perceived Stress Scale^59^ were included to assess symptoms of depression and anxiety and recent perceived stress.

### Childhood adversity

Participant interview was used to elicit the history of loss of a parent before the age of 18 (*N*=88). This category included death (*n*=38) of a parent and/or prolonged separation/desertion (that is, parent deserted for at least 6 months with no attempts at contact or responses to child's attempts, *n*=51).

The 28-item Childhood Trauma Questionnaire^60^ is a self-report measure that generates a total score summarizing five types of childhood maltreatment (physical, sexual and emotional abuse, physical and emotional neglect), with high internal consistency, test–retest reliability and convergent validity.^60, 61, 62, 63, 64, 65^ Threshold scores corresponding with 'moderate' or 'severe' exposure on each maltreatment subscale were used to define the presence of each adversity type on this scale.^63^ Adversity was considered present if the score was greater than or equal to 13 for emotional abuse, 10 for physical abuse, 8 for sexual abuse, 10 for physical neglect and 15 for emotional neglect.

Participants who experienced parental death or desertion or at least one of the maltreatment types assessed by the Childhood Trauma Questionnaire were considered to have experienced childhood adversity. In addition, an index of early stress exposure was created by summing the number of adversities (parental death, parental desertion and the five types of childhood maltreatment on the Childhood Trauma Questionnaire).

### NR3C1 promoter methylation sequencing

Whole blood was frozen within 20 min of blood draw at −80 °C until processing. Samples were labeled with a numerical code only, and laboratory personnel were blind to subject characteristics. DNA was extracted from frozen whole blood using PAXgene reagents (Qiagen, Valencia, CA, USA) according to the manufacturer's directions. Methylation at the *NR3C1* promoter region was examined with a quantitative pyrosequencing approach following the method of Oberlander and colleagues as previously described.^31, 36^ The region analyzed contains 13 CpGs and encompasses exon 1F, the human homolog of the rat exon 1_7_. Sodium bisulfite modification of 500 ng of DNA was performed using the EZ DNA Methylation Kit (Zymo Research, Orange, CA, USA) following the manufacturer's protocol. For quality control, PCR products were visualized and sized on an agarose gel (FlashGel, Lonza, Basel, Switzerland); samples were run in triplicate for pyrosequencing and data points were rejected if they were greater or less than two times the s.d. of the mean of the triplicate group. Peripheral blood lymphocyte DNA that was not sodium bisulfite-modified was included in each pyrosequencing run and served as a control for nonspecific amplification. Methylation quantification was performed using the Pyromark Software (Qiagen). The percent of alleles that were methylated in the cell population examined was used in statistical analyses.

### Dex/CRH test

On a subsequent visit, 231 of the participants completed the Dex/CRH test. The night before the test, an oral dose of dexamethasone 1.5 mg was self-administered at 2300 hours. The following day, participants arrived at 1200 hours and were given lunch. A topical anesthetic (lidocaine 2.5% and prilocaine 2.5%) was applied to the participant's forearm between 1230 and 1245 hours. At 1300 hours an indwelling intravenous catheter was inserted in the forearm by a highly experienced research nurse. Participants then remained in a semi-recumbent position throughout the procedure except to use the bathroom. They were permitted to read or watch pre-selected materials that did not contain emotionally charged material. Vital signs were monitored throughout the test. At 1500 hours, CRH 100 μg (corticorelin ovine triflutate, Acthrel, Ferring Pharmaceuticals, Parsippany, NJ, USA) reconstituted in 2 ml of 0.9% NaCl solution was infused intravenously over 30 s. Blood samples were drawn at 1459, 1530, 1545, 1600, 1615 and 1700 hours and assayed for cortisol. The 1459 hours sample represents the response to dexamethasone (post-Dex) before administration of CRH infusion.

### Hormone assays

Plasma cortisol concentrations were assayed in duplicate using the following assays and following the manufacturers' instructions. Samples were labeled with a numerical code only and laboratory personnel were blind to subject characteristics. The Gamma Coat cortisol I-125 coated-tube radioimmunoassay kit (INCSTAR, Stillwater, MN, USA) was used for 106 participants. Intra- and interassay CVs observed for quality assessment samples (5 and 20 μg /dl) were less than 5% and 10%, respectively. The double antibody DSL-2000 Cortisol Radioimmunoassay Kit (Diagnostic Systems Laboratories, Webster, TX, USA) was used for 81 participants. The intra- and interassay coefficients of variation are 5.3% and 7.0%, respectively. The cortisol Chemiluminescence Immunoassay kit (Beckman-Coulter, Brea, CA, USA) was used for 44 participants. The intra- and interassay coefficients of variation are both 4.3%. Variation between these three assay types was assessed in *n*=19 samples that were analyzed using more than one assay type, and results showed very high correlations (*r*=0.92–0.98). Area under the curve (AUC) over time for cortisol response to the Dex/CRH test was calculated using the trapezoidal rule. Post-Dex and AUC values were log-transformed because of positive skew.

### Statistical analysis

In order to test hypothesized effects of early stress and psychopathology, four groups were examined: participants with no early adversity and no lifetime psychiatric diagnosis (*n*=96, No Adversity/No Disorder group), participants with early adversity but no lifetime diagnosis (*n*=60, Adversity/No Disorder group), participants with a lifetime diagnosis but no history of early adversity (*n*=51, No Adversity/Disorder group) and those with both early adversity and a lifetime diagnosis (*n*=133, Adversity/Disorder group). Subsequent analyses examined effects of specific disorder categories (lifetime MDD, lifetime depressive disorder (including MDD), lifetime PTSD, lifetime anxiety disorder (including PTSD) and past substance-use disorder) and childhood adversity types (physical abuse, sexual abuse, emotional abuse, physical neglect, emotional neglect, parental death and parental desertion). Effects of the number of adversities on methylation were also examined.

Our primary analyses examined effects of the adversity/disorder grouping on mean percent methylation of CpG sites across the 1_F_ region. Methylation was correlated across CpG sites in this region. As with our prior work, CpG 7–13 were most highly intercorrelated (*r*=0.72-0.91) and CpG 1–6 were more modestly intercorrelated (*r*=0.42–0.66). Correlations across CpG 1–6 and CpG 7–13 were *r*=0.33–0.78. Given this intercorrelation, we examined mean methylation across the entire region, and, in the case of a significant overall effect on mean methylation, we then conducted follow-up analyses to examine individual CpG sites. We did not adjust for multiple comparisons because individual sites were only examined as *post hoc* comparisons in the event of a significant omnibus test. Outliers, defined as values more than three s.d.'s from the mean, were Winsorized by setting them to the next highest value within three s.d.'s. The number of outliers at each individual CpG site ranged from 1 (CpG 5) to 9 (CpG 1), with a mean of 5.4 outliers at each site (1.6% of the sample).

Analyses were conducted with SPSS version 20 (IBM, Armonk, NY, USA). All analyses were two-tailed with alpha=0.05 and data conformed to the assumptions of the statistical tests. Levene's test of the homogeneity of variance demonstrated that for mean methylation across the 13 CpG sites, the variances were equal across the four Adversity/Disorder groups (F(3, 336)=1.27, *P*=0.286). With *N*=340, we have power ≥0.80 to detect effect sizes as small as *f*=0.18, a small-medium-sized effect.^66^ Demographic characteristics, as well as recent perceived stress levels and subsyndromal symptoms of depression and anxiety, were examined and controlled where appropriate. General linear models, controlling for relevant covariates, were used to test for effects of adversity and psychopathology. Partial correlations, controlling for age, sex and oral contraceptive use, were used to examine associations of *NR3C1* methylation with post-Dex and AUC cortisol in the Dex/CRH test. Error bars in figures represent s.e.m.

## Results

### Preliminary analyses

Participant characteristics are displayed in Table 1. One hundred and ninety-three participants reported a history of early adversity; 184 had a past substance-use disorder or a current or lifetime depressive or anxiety disorder (see table for types of adversity and disorders). Participants in the Adversity/Disorder group were significantly older than those in the other groups (Table 1), and age was associated with methylation at CpG 1 (*r*=0.13, *P*=0.020) and CpG 2 (*r*=0.13, *P*=0.014). Gender did not differ according to group; however, women had greater methylation at CpG 3 than men (*t*=−2.01, *P*=0.046). To control for these associations, age and gender were included as covariates in models testing associations of the Adversity/Disorder groupings and *NR3C1* methylation. The Adversity/Disorder groups differed with respect to the proportion of participants who identified their race as white versus those who identified another race (Table 1). However, there were no differences in *NR3C1* methylation at any of the CpG sites based on race, and associations of the Adversity/Disorder groupings and the number of adversities with *NR3C1* did not differ when race was included in the models; therefore, it was not further considered. There was no difference in mean methylation across the 13 CpG sites between women with and without use of oral contraceptives. For analyses testing associations of the Adversity/Disorder grouping and the Dex/CRH test response, use of oral contraceptives was associated with greater post-dexamethasone cortisol (*t=−*6.19, *P*<0.001) and greater cortisol at 120 min following CRH administration (*t=−*2.03, *P*=0.043); this variable was controlled in addition to age and gender.

### Associations of Adversity/Disorder grouping variable with NR3C1 methylation

**Figure 1:**
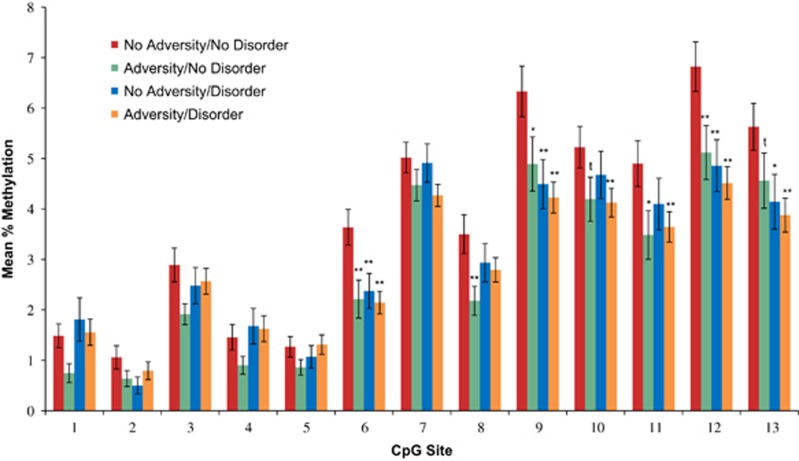
Group differences in methylation at individual *NR3C1* CpG sites Note. ^t^*P*<0.10; *P<0.05; **P<0.01.

The Adversity/Disorder grouping variable was associated with mean methylation across the 13 CpG sites (F(3, 334)=3.68, *P*=0.012). The No Adversity/No Disorder group had significantly higher levels of mean methylation than the Adversity/No Disorder group (*P*=0.013) and the Adversity/Disorder group (*P*=0.002), and this difference was at the trend level for the No Adversity/Disorder group (*P*=0.06). *Post hoc* examination of individual CpG sites (Figure 1) revealed that there were significant differences between the four groups at CpG 6 (F=6.65, *P*<0.001), CpG 9 (F=6.28, *P*<0.001), CpG 10 (F=2.70, *P*=0.046), CpG 11 (F=2.82, *P*=0.039), CpG 12 (F=7.35, *P*<0.001) and CpG 13 (F=4.18, *P*=0.006), and a trend-level effect at CpG 8 (F=2.39, *P*=0.069). The No Adversity/No Disorder group had the highest levels of methylation, and this was the only group to differ significantly from any other group. The two Adversity groups showed the most consistent differences across this region, and, in addition, the No Adversity/Disorder group also had significantly lower levels of methylation at CpGs 6, 9, 12 and 13.

### Analysis of diagnostic categories

Participants with any lifetime depressive disorder (F=7.54, *P*=0.007, *n*=115), any lifetime anxiety disorder (F=5.89, *P*=0.017, *n*=40) and any past substance-use disorder (F=3.89, *P*=0.050, *n*=75) had lower levels of methylation than those in the No Adversity/No Disorder group (*n*=96) across the 13 CpG sites. Table 2 shows results of *post hoc* analyses of associations of individual CpG sites with these disorders. There was no significant difference in mean methylation between those with No Adversity/No Disorder and those with lifetime MDD (*P*=0.132, *n*=97) or those with lifetime PTSD (*P*=0.422, *n*=16).

### Analysis of adversity types

Participants who experienced emotional abuse (F=6.45, *P*=0.012, *n*=83), emotional neglect (F=6.63, *P*=0.011, *n*=88), physical neglect (F=7.19, *P*=0.008, *n*=55) and parental desertion (F=6.99, *P*=0.009, *n*=51) had lower levels of methylation than those in the No Adversity/No Disorder group across the 13 CpG sites. Table 2 shows results of *post hoc* analyses of associations of individual CpG sites with these adversity types. The reduced mean methylation in those with physical abuse (*n*=58), sexual abuse (*n*=63) and parental death (*n*=38) in comparison with those in the No Adversity/No Disorder group (*n*=96) did not reach significance (*P*=0.15, 0.17 and 0.11, respectively).

### Associations of the number of adversities with NR3C1 methylation

The number of adversities was negatively associated with mean methylation across the 13 CpG sites (*r*=−0.11, *P*=0.046), and *post hoc* analyses of individual sites showed significant negative associations with CpGs, 6, 7, 9, 10, 11, 12 and 13 (*P-*values<0.05). Participants with disorders (lifetime depressive and anxiety disorders and past substance-use disorders) had a greater number of total adversities and were more likely to have each type of maltreatment (Table 1). There was a significant interaction of the number of adversities and lifetime psychiatric disorder (F(1, 334)=5.84, *P*=0.016), such that number of adversities was negatively associated with mean methylation across the region in participants with no lifetime disorder (*r*=−0.19, *P*=0.017), but not in those with a lifetime disorder (*r*=−0.03, not significant). *Post hoc* examination of the individual CpG sites revealed that this effect was significant for CpG 2 (F=4.50, *P*=0.035), CpG 3 (F=6.92, *P*=0.009), CpG 6 (F=4.00, *P*=0.046), CpG 8 (F=5.12, *P*=0.024), CpG 10 (F=4.30, *P*=0.039), CpG 11 (F=4.60, *P*=0.033) and CpG 12 (F=4.22, *P*=0.041). There were trend-level effects of the interaction on methylation at CpGs 1, 9 and 13 (*P*=0.085, 0.057 and 0.092, respectively). Consistent with the effect for mean methylation across the 13 CpG sites, the number of adversities was negatively associated with individual CpG site methylation in those participants without psychopathology (*r*'s range from −0.14 to −0.19, *P*'s range from 0.02 to 0.09), but was not associated with methylation at the individual CpG sites in participants with lifetime psychiatric disorders (*r*'s range from −0.06 to 0.07, *P*'s range from 0.35 to 0.89).

### Sensitivity analyses

As shown in Table 1, there were significant differences between the four groups in perceived stress (F=20.13, *P*<0.001), depressive symptoms (F=44.10, *P*<0.001), state-related anxiety symptoms (F=17.27, *P*<0.001) and trait-related anxiety symptoms (F=26.40, *P*<0.001). Mean methylation across the 13 CpG sites was negatively associated with trait-related anxiety symptoms and with perceived stress at the trend level (*r*=−0.11, *P*=0.057; *r*=−0.11, *P*=0.066, respectively), but not with depressive symptoms or state-related anxiety symptoms. When added to the model assessing effects of the Adversity/Disorder grouping, none of these variables was significantly associated with mean methylation across the 13 CpG sites, and the Adversity/Disorder grouping variable remained a significant predictor of *NR3C1* methylation.

### Cortisol response to the Dex/CRH test

**Figure 2:**
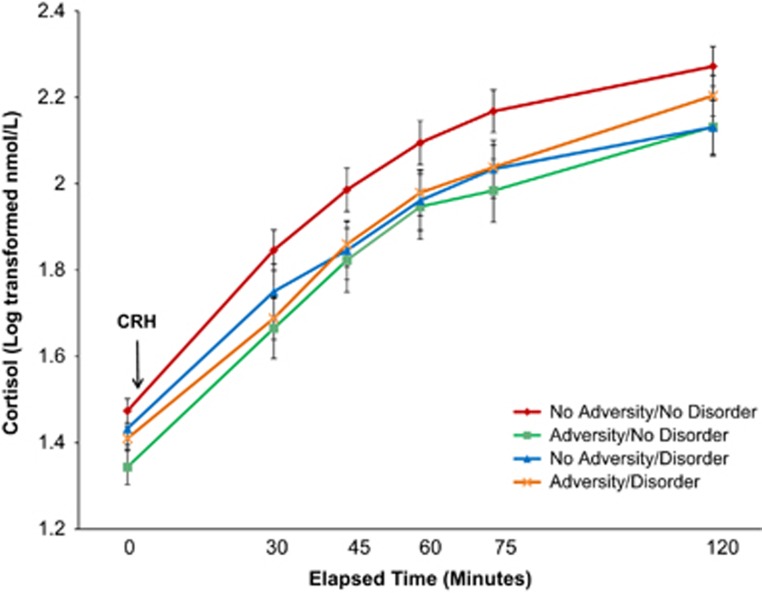
Cortisol response to dexamethasone/corticotropin-releasing hormone (Dex/CRH) test in relation to adversity/disorder grouping. Note: the general linear model testing the effect of group on cortisol response over time did not reach significance (F(3, 220)=2.08, *P*=0.103).

Cortisol response to the Dex/CRH test according to group is shown in Figure 2. Although the No Adversity/No Disorder group had the highest cortisol concentrations in the test, this did not reach significance (F(3, 220)=2.08, *P*=0.103). Similarly, effects of the Adversity/Disorder grouping did not reach significance for post-Dex cortisol (F(3, 222)=2.48, *P*=0.062) or cortisol AUC (F(3, 221)=2.23, *P*=0.086).

Associations of *NR3C1* methylation and cortisol response to the Dex/CRH test are displayed in Table 3. Mean methylation across the 13 sites was positively associated with post-Dex cortisol (*r*=0.148, *P*<0.05). *Post hoc* analyses of individual CpG sites showed that post-Dex cortisol had significant positive associations with methylation at CpG 2, CpG 5, CpG 7, CpG 9 and CpG 12 (*P*<0.05) and CpG 1 and CpG 3 (*P*<0.01), and there was a trend-level association with CpG 6, CpG 8, CpG 11 and CpG 13 (*P*<0.10). Cortisol AUC was associated with mean methylation across the 13 CpG sites at the trend level (*P*<0.10), and exploratory analyses at individual CpG sites showed significant effects at CpG 8 and CpG 11 (*P*<0.05), and at trend level for CpG 6 and 7 (*P*<0.10).

## Discussion

In this study we found that adults with a history of childhood adversity had reduced levels of methylation in exon 1_F_ of the promoter of the GR gene. In addition to lower levels of methylation across the region, the groups with early adversity showed lower methylation at several individual CpG sites. The group with lifetime depressive, anxiety and substance-use disorders who did not have a history of childhood adversity had trend-level reductions in mean methylation across this region, with a few of the individual CpG sites showing significantly lower methylation in this group. Consistent with the expectation that lower methylation would be associated with greater GR numbers and enhanced glucocorticoid negative feedback, lower methylation at several sites in this region was associated with lower post-Dex cortisol responses to the Dex/CRH test. Those with early adversity and/or psychopathology tended to have lower cortisol responses to the test, although this was not statistically significant (in the subset of the sample that participated in the Dex/CRH test).

These findings stand in contrast to prior work that has documented higher levels of methylation of this region of *NR3C1* with early stress.^27, 31, 35, 36, 38, 48^ However, some studies have found reduced methylation at individual CpG sites in *NR3C1* 1_F_ in association with early stress.^28, 35, 38^ In addition, although two studies of subjects with internalizing symptoms have shown increased NR3C1 methylation as compared with controls,^46, 53^ there is evidence of decreased methylation at two CpG sites within the 1F region among patients with depression.^42^ Reduced levels of methylation in this region have also been reported in studies of patients with PTSD,^44, 45, 54^ and externalizing disorders.^40^ Individuals using psychotropic medication were excluded from the present study. It is important to note that most of the prior work on *NR3C1* methylation in patients with psychiatric conditions did not exclude patients on psychotropic medication that can alter methylation levels.^48, 49, 50^ Our finding of reduced levels of methylation could be due to a loss of methyl groups over time, or inhibition of methylation in response to stress and trauma. In rats, the region of *NR3C1* homologous to the 1_F_ promoter is epigenetically regulated in response to chronic stress exposure in adulthood.^67^ Thus, it is possible that some of the group differences we observed may have occurred in response to experiences occurring over time in adulthood; however, it is important to note that effects of current symptoms or perceived stress did not account for our group findings.

We believe this is the first study to examine *NR3C1* methylation in adults with and without a history of childhood adversity and with and without depressive, anxiety or substance-use disorders. The groups with early adversity, with or without psychopathology, had significantly lower methylation across the 13 sites and at several individual CpG sites. Participants with lifetime depressive, anxiety or substance-use disorders but no childhood adversity had numerically lower mean methylation across this region; however, this contrast did not reach statistical significance. *Post hoc* examination of individual CpG sites suggested that the group with psychopathology alone had lower methylation of some, but not all, of the sites that were significant for early adversity. The group with *both* adversity and lifetime psychopathology tended to show the greatest difference from controls; however, it should be noted that this group had more adverse early experiences, and that methylation in this group did not differ significantly from those with adversity but without lifetime psychopathology. The number of adversities was associated with lower methylation across the region and at several individual CpG sites in those without lifetime disorders, suggesting a dose effect. Taken together, these findings suggest that childhood adversity influences *NR3C1* methylation and may represent a risk factor for the development of psychopathology. Psychiatric disorders may contribute to alterations in methylation of *NR3C1* to a lesser degree, possibly via effects of these disorders on behavioral and physiological factors involved in stress responses.

We also examined the contributions of specific types of adversity and disorders. All adversity types were associated with lower methylation, and adversity types that tend to be chronic, including emotional abuse, emotional neglect, physical neglect and parental desertion were statistically significant. In contrast, physical abuse, sexual abuse and parental death did not reach statistical significance; these forms of adversity may be less pervasive and chronic than the other types, and children with such experiences may also experience buffering effects of positive emotional influences. Turning to individual disorder types, lifetime depressive disorder, lifetime anxiety disorder and past substance abuse showed significant effects, whereas lifetime PTSD did not, and lifetime MDD did not reach significance. However, it is important to note that lifetime MDD (*n*=97) was included in the significant effect for the lifetime depressive disorder (*N*=115) group. The number of participants with lifetime PTSD was small (*n*=16), limiting our power to detect an effect.

In our prior preliminary work with a sample of 99 healthy adults with no lifetime psychopathology, we found that early adversity was associated with greater methylation in this region of *NR3C1*. Although the same technique was used to measure methylation in both samples, methylation levels in the current sample were lower overall in comparison with the prior sample. The previous sample was younger, with a mean age of 27, and there may be loss of methylation with age;^68, 69^ however, we did control for age in the analyses. In our prior study, effects of early adversity were seen at CpGs 1–4, a region where methylation was particularly low in the current sample; therefore, there may be a 'floor effect'. In the present study, hypomethylation with adversity and psychopathology occurred in regions with overall higher levels of methylation. With respect to cortisol responses to the Dex/CRH test, in our prior study we found attenuated cortisol responses in association with higher methylation at CpG 2 and in exploratory analyses of sites CpG 5 and 7–13. We noted that this contrasted with prior work and seemed unlikely to reflect a direct effect of methylation-induced decreases in GR expression. In this much larger study of individuals with and without early adversity and with and without certain lifetime disorders, we find small but significant positive associations of post-dexamethasone cortisol with mean methylation and at several CpG sites across this region, as well as a trend for cortisol AUC. The direction of this effect is in keeping with findings of most, but not all, other studies,^26^ and with the expectation that lower methylation would be associated with increased gene expression and thus with enhanced HPA-axis negative feedback and reduced cortisol responses. These results from our study and others suggest that methylation of *NR3C1* and its relationship to HPA-axis function is complex. It is possible that other factors such as participant age at the time of exposure to critical stressors, unmeasured adverse experiences, chronicity or severity of stressors, methylation of other HPA-regulatory genes or other factors inherent to depressive, anxiety and substance-use disorders also influenced *NR3C1* methylation.^26^

Differential methylation of key TF-binding sites within the promoter regions is one mechanism by which adversity could exert influence on *NR3C1* expression. The 1_F_ region of *NR3C1* contains binding sites for the nerve growth factor-inducible protein A (NGFI-A) that increases expression of GR, an effect that can be inhibited by methylation of these sites in both humans and the homologous 1_7_ region in rodents.^23, 38^ Several,^23, 24, 25, 26, 31, 32, 36, 70^ but not all^27, 28, 29, 33^ studies have found increased methylation at the canonical NGFI-A-binding site (CpG 3 and CpG 4 in the present study), in humans and rodents with early adverse environmental exposures; this may have a functional effect on HPA-axis activity.^28, 31, 71^ Other studies have also reported a positive association between adversity and methylation of other canonical and non-canonical NGFI-A-binding sites within the 1F region.^29, 38, 53^ Decreased methylation within the NGFI-A-binding site has been documented in MDD^42^ and PTSD.^42, 45^ In the present study we did not find an effect of the adversity/disorder grouping on methylation at this binding site; however, the number of adversities was negatively linked to methylation at CpG 3 in those with no psychopathology, and there was a significant positive correlation between CpG 3 methylation and post-dexamethasone cortisol.

Some evidence indicates that interference with NGFI-A binding is not sufficient to reduce GR transcription and that methylation of other TF-binding sites across the 1_F_ region may be involved.^67^ Armstrong *et al.*^51^ ran a sequence-based *in silico* query to identify other possible transcription factor-binding sites within *NR3C1* 1_F_. Numerous potential binding sites were identified, including sites for the alcohol dehydrogenase regulator 1, specificity protein 1 and heat shock factor.^51^ Some of these factors, such as heat shock factor that lies in proximity to CpG 9, are known to be involved in immune and stress responses.^72^ Consistent with this, CpG 9 showed differential methylation for all adversity types and diagnostic categories in this study. Putative binding sites for alcohol dehydrogenase regulator 1 reside in proximity to CpGs 4, 10, 11 and 13, which showed differential methylation with many adversity and diagnostic categories in our analyses. Putative sites for specificity protein 1 lie in proximity to CpGs 7 and 12, units that also showed differential methylation with several of our adversity and diagnostic categories. Differential methylation of CpGs within these binding sites may interfere with TF binding and regulate GR expression in response to adversity.

However, it is important to note that, consistent with findings from numerous research laboratories,^8, 31, 40, 42, 45, 47, 53, 73, 74^ levels of *NR3C1* exon 1_F_ methylation in this study were quite low, and may not be high enough to interfere with TF binding. Low levels of methylation typically characterize regions of the genome that are open to regulation by methylation.^22, 75, 76^ In contrast, high methylation is seen in areas where CpG density is low and transcription is not usually initiated.^75^ Our investigation was limited by the lack of gene expression data, but our finding linking lower levels of methylation at several CpG sites to lower Dex/CRH cortisol responses is consistent with prior work^45, 77^ and suggests that reduced methylation could have a functional effect on expression of GR.

This study is limited by the use of whole blood containing DNA from multiple different cell types. It is possible that our results were affected by differences in methylation across leukocyte types that might co-vary with adversity and psychopathology. We attempted to minimize such effects by excluding participants with acute or chronic illness or pregnancy. Our use of peripheral blood limits our ability to draw conclusions about central processes. However, peripheral and central DNA methylation and expression is likely to be more highly co-regulated for systems that have widespread effects, such as the glucocorticoid and immune systems.^73, 74^ We did not assess lifetime externalizing disorders; recent work suggests that externalizing disorders are associated with a decrease in *NR3C1* methylation levels;^40^ therefore, such a history may contribute to the methylation patterns observed in this study. Other limitations include the cross-sectional and retrospective nature of the study and adversity data, the inclusion of participants taking oral contraceptives and the lack of control for menstrual phase in females. However, in contrast to most studies of *NR3C1* methylation in psychiatric conditions, a major strength of this study is that we excluded individuals taking other medications as well as individuals with acute or chronic medical conditions. Finally, it should be noted that we did not include examination of additional genes or markers of global methylation. Epigenetic changes to other genes that are involved in the response to adversity and risk for psychopathology and other poor health outcomes^78, 79, 80^ should be considered in future work.

In conclusion, our findings of lower levels of methylation of the 1_F_ promoter region of *NR3C1* in relation to early stress and psychiatric conditions that are often associated with stress exposure suggest that these relationships are more complex than previously understood. Future work is needed to replicate these findings and clarify the mechanisms and sequelae of differential methylation of *NR3C1* in association with early stress and psychopathology.

## Figures and Tables

**Table 1:** Adversity/Disorder grouping variable descriptives

	*No Adversity/No Disorder (*n*=96)*	*Adversity/No Disorder (*n*=60)*	*No Adversity/Disorder (*n*=51)*	*Adversity/Disorder (*n*=133)*	P
Age *M* (s.d.)	29.60 (10.04)^a^	31.90 (10.19)^a^	32.08 (11.29)^a^	35.95 (12.37)^b^	0.00
Sex *N* (%) male	38 (39.6)	22 (36.7)	21 (41.2)	46 (34.6)	0.81
Race *N* (%) white	74 (77.1)^a^	37 (61.7)^b^	46 (90.2)^a^	108 (81.2)^a^	0.00
Education *N* (%), ≥ college degree	54 (56.3)	24 (40.0)	23 (45.1)	44 (33.1)	0.35
Emotional abuse *N* (%)	—	15 (25.0)^a^	-—	68 (51.1)^b^	0.00
Physical abuse *N* (%)	—	10 (16.7)^a^	—	48 (36.1)^b^	0.01
Sexual abuse *N* (%)	—	13 (21.7)^a^	—	50 (37.6)^b^	0.03
Emotional neglect *N* (%)	—	16 (26.7)^a^	—	72 (54.1)^b^	0.00
Physical neglect *N* (%)	—	10 (16.7)^a^	—	45 (33.8)^b^	0.02
Parental death *N* (%)	—	15 (25.0)	—	23 (17.3)	0.26
Parental desertion *N* (%)	—	21 (35.0)	—	30 (22.6)	0.13
Number of adversities *M* (s.d.)	—	1.73 (1.16)^a^	—	2.62 (1.51)^b^	0.00
Current MDD *N* (%)	—	—	9 (17.6)^a^	44 (33.1)^b^	0.04
Past MDD *N* (%)	—	—	18 (35.3)	43 (32.3)	0.70
Current dysthymia/Dep NOS *N* (%)	—	—	4 (7.8)	16 (12.0)	0.41
Past dysthymia/Dep NOS *N* (%)	—	—	4 (7.8)	20 (15.0)	0.20
Current PTSD *N* (%)	—	—	0 (0.0)	4 (3.0)	0.21
Past PTSD *N* (%)	—	—	2 (3.9)	11 (8.3)	0.30
Current social phobia *N* (%)	—	—	2 (3.9)	7 (5.3)	0.71
Past social phobia *N* (%)	—	—	1 (2.0)	5 (3.8)	0.54
Current other anxiety disorder *N* (%)	—	—	4 (7.8)	4 (3.0)	0.15
Past other anxiety disorder *N* (%)	—	—	2 (3.9)	11 (8.3)	0.30
Past substance disorder *N* (%)	—	—	27 (52.9)^a^	48 (36.1)^b^	0.04
IDSSR *M* (s.d.)	6.39 (5.90)^a^	9.29 (6.69)^a^	16.43 (12.87)^b^	21.81 (13.57)^c^	0.00
STAI—Trait *M* (s.d.)	27.48 (7.28)^a^	31.43 (9.79)^b^	38.14 (12.55)^c^	39.41 (11.27)^c^	0.00
STAI—State *M* (s.d.)	26.56 (6.94)^a^	27.95 (7.13)^a^	35.32 (12.98)^b^	34.43 (10.10)^b^	0.00
PSS *M* (s.d.)	16.33 (5.67)^a^	18.89 (6.81)^b^	23.88 (9.64)^c^	23.83 (8.47)^c^	0.00

Abbreviations: ANOVA, analysis of variance; Dep, depression; IDSSR, Inventory of Depressive Symptomatology, Self-Report; MDD, major depressive disorder; NOS, not otherwise specified; PTSD, post-traumatic stress disorder; STAI, State-Trait Anxiety Inventory.

*Note*: In four group analyses, *P*-values indicate ANOVA significance level. In two group analyses, *P*-values indicate *t-*test and *?*^2^-significance level. Different superscripts within the same line indicate groups are significantly different from one another at *P*<0.05. Other anxiety disorders include generalized anxiety disorder, panic disorder, agoraphobia and anxiety disorder NOS.

**Table 2:** CpG site-specific methylation for significant disorder and adversity types in comparison with No Adversity/No Disorder group

*CpG site*	*Depressive Disorder (*n*=115)*	*Anxiety Disorder (*n*=40)*	*Past Substance Disorder (*n*=75)*	*Emotional Abuse (*n*=83)*	*Emotional Neglect (*n*=88)*	*Physical Neglect (*n*=55)*	*Parental Desertion (*n*=51)*
Mean	7.54**	5.89*	3.89*	6.45*	6.63*	7.19**	6.99**
1	ns	ns	ns	ns	ns	ns	ns
2	ns	ns	ns	ns	ns	ns	ns
3	ns	ns	ns	ns	ns	ns	ns
4	ns	ns	ns	ns	ns	ns	ns
5	ns	ns	ns	ns	ns	ns	ns
6	14.56***	11.75***	7.40**	8.02**	11.60***	8.65**	12.67***
7	3.44^t^	5.08*	ns	3.59^t^	3.38^t^	4.37*	ns
8	ns	ns	ns	3.49^t^	ns	3.15^t^	3.34^t^
9	19.56***	14.05***	8.25**	11.02***	12.35***	12.71***	8.77**
10	6.09*	5.28*	ns	6.06*	5.33*	9.47**	5.18*
11	6.07*	5.59*	3.15^t^	5.76*	4.84*	6.96**	5.79*
12	21.02***	13.01***	8.64**	14.03***	15.94***	17.66***	11.24***
13	12.48***	12.47***	7.03**	7.76**	8.07**	10.33**	5.68*

Abbreviation: ns, not significant.

*Note*: All table values represent the F statistic. ^t^*P*<0.10, *P<0.05, **P<0.01, ***P<0.001.

**Table 3:** Associations of *NR3C1* methylation at individual CpG sites and cortisol concentrations in the Dex/CRH test

	*Mean CpG 1 - 13*	*CpG 1*	*CpG 2*	*CpG 3*	*CpG 4*	*CpG 5*	*CpG 6*	*CpG 7*	*CpG 8*	*CpG 9*	*CpG 10*	*CpG 11*	*CpG 12*	*CpG 13*
Post-Dex	0.148*	0.209**	0.136*	0.206^**^	0.106	0.147*	0.113^t^	0.162*	0.118^t^	0.155*	0.098	0.122^t^	0.143*	0.126^t^
AUC	0.117^t^	0.056	0.028	0.083	0.026	0.058	0.122^t^	0.114^t^	0.166*	0.098	0.095	0.139*	0.093	0.099

Abbreviations: AUC, area under the curve; Dex/CRH, dexamethasone/corticotropin-releasing hormone.

Note: values indicate Pearson's Coefficient; ^t^*P*<0.10, *P<0.05, **P<0.01.
